# Association between *Helicobacter pylori* seropositivity and the hemoglobin A1c/high-density lipoprotein cholesterol ratio in U.S. adults: evidence from NHANES

**DOI:** 10.3389/fnut.2025.1589510

**Published:** 2025-06-09

**Authors:** Cheng Xu, Xin-yi Jiang, Jia-ming Liao, Yi-fan Zhao, Jing-yi Hu, Chong-Chao Li, Hong Shen

**Affiliations:** ^1^The First Clinical College, Nanjing University of Chinese Medicine, Nanjing, China; ^2^Affiliated Hospital of Nanjing University of Chinese Medicine, Nanjing, China; ^3^Institute of Literature in Chinese Medicine, Nanjing University of Chinese Medicine, Nanjing, China

**Keywords:** *Helicobacter pylori*, hemoglobin A1c/high-density lipoprotein cholesterol ratio, metabolic dysfunction, NHANES, cross-sectional study

## Abstract

**Background:**

*Helicobacter pylori* (*H. pylori*) infection is associated with insulin resistance and metabolic syndrome. This study investigates the association between *H. pylori* seropositivity and the newly proposed hemoglobin A1c/high-density lipoprotein cholesterol ratio (HbA1c/HDL-C ratio) in a nationally representative U.S. population.

**Methods:**

Data from the 1999–2000 National Health and Nutrition Examination Survey (NHANES) were analyzed. Multivariable linear regression models assessed the association between *H. pylori* seropositivity and the HbA1c/HDL-C ratio. Subgroup analyses were performed to evaluate the consistency of the association across different demographic and clinical strata. Generalized additive models with smoothing splines and threshold effect analysis was conducted to identify potential nonlinear relationships.

**Results:**

The cross-sectional analysis comprised 2,909 participants, including 1,254 with *H. pylori* seropositivity. After multivariable adjustment, a significant positive association was found between *H. pylori* seropositivity and the HbA1c/HDL-C ratio (*β*: 0.28, 95% CI: 0.13, 0.42). Subgroup analyses revealed a stronger association among non-diabetic individuals compared to diabetic individuals. A “L”-shaped relationship was observed, with an inflection point at an HbA1c/HDL-C ratio of 4.81. Below this threshold, *H. pylori* seropositivity was positively associated with the HbA1c/HDL-C ratio. Above this threshold, the association was no longer statistically significant.

**Conclusion:**

This study identifies a significant association between *H. pylori* seropositivity and the HbA1c/HDL-C ratio, suggesting that metabolic dysfunction may be linked to *H. pylori* infection. Future longitudinal studies are needed to establish causality and explore underlying mechanisms.

## 1 Introduction

*Helicobacter pylori* (*H. pylori*) infection is one of the most prevalent chronic bacterial infections in the world, affecting about half of the global population (1, 2). It is a major cause of several gastrointestinal disorders, including chronic gastritis, peptic ulcer and gastric cancer (3, 4). In addition, many extra-gastric diseases have been shown to be associated with *H. pylori* infection, such as metabolic, cardiovascular and neurological disorders (5–7).

Emerging evidence suggests that *H. pylori* infection may contribute to systemic metabolic disturbances, including insulin resistance (IR) and metabolic syndrome (8–10). This association appears to be bidirectional, with individuals who already exhibit metabolic disturbances being more prone to persistent *H. pylori* infection (11). However, the existing literature presents inconsistent results, underscoring the necessity for novel biomarkers to disentangle this intricate interplay. In recent years, the HbA1c/HDL-C ratio has been introduced as a comprehensive indicator of both glucose and lipid metabolism. Hemoglobin A1c (HbA1c), a marker reflecting average blood glucose levels over the past 2–3 months, provides insight into long-term glycemic control (12, 13). On the other hand, high-density lipoprotein cholesterol (HDL-C), known for its anti-inflammatory and antioxidant properties, plays a key role in lipid metabolism and is generally regarded as a protective factor against cardiovascular diseases (14). Thus, the HbA1c/HDL-C ratio serves as a refined measure of the interplay between glucose regulation and lipid balance, offering valuable information about overall metabolic state. This ratio has gained considerable attention for its potential in assessing the risk of various conditions, including carotid atherosclerosis (15), stroke (16), and metabolic associated fatty liver disease (17). Despite its promising clinical significance, no studies have yet examined the relationship between the HbA1c/HDL-C ratio and *H. pylori* infection.

This study aims to investigate the association between *H. pylori* seropositivity and the HbA1c/HDL-C ratio in a nationally representative sample of U.S. adults. By exploring this relationship, we seek to identify potential metabolic markers for *H. pylori* susceptibility and provide new insights into the interplay between metabolic health and infectious diseases.

## 2 Methods

### 2.1 Study design and population

The National Health and Nutrition Examination Survey (NHANES) is a nationally representative, cross-sectional program designed to assess the health and nutritional status of the non-institutionalized civilian population in the U.S. (18, 19). Employing a complex, multi-stage probability sampling design, NHANES collects data through structured interviews, standardized physical examinations, and comprehensive laboratory analyses, providing a robust platform for evaluating a wide range of health indicators (19–21). All study protocols were reviewed and approved by the Ethics Review Board of the National Center for Health Statistics, and written informed consent was obtained from all participants.

**Figure 1:**
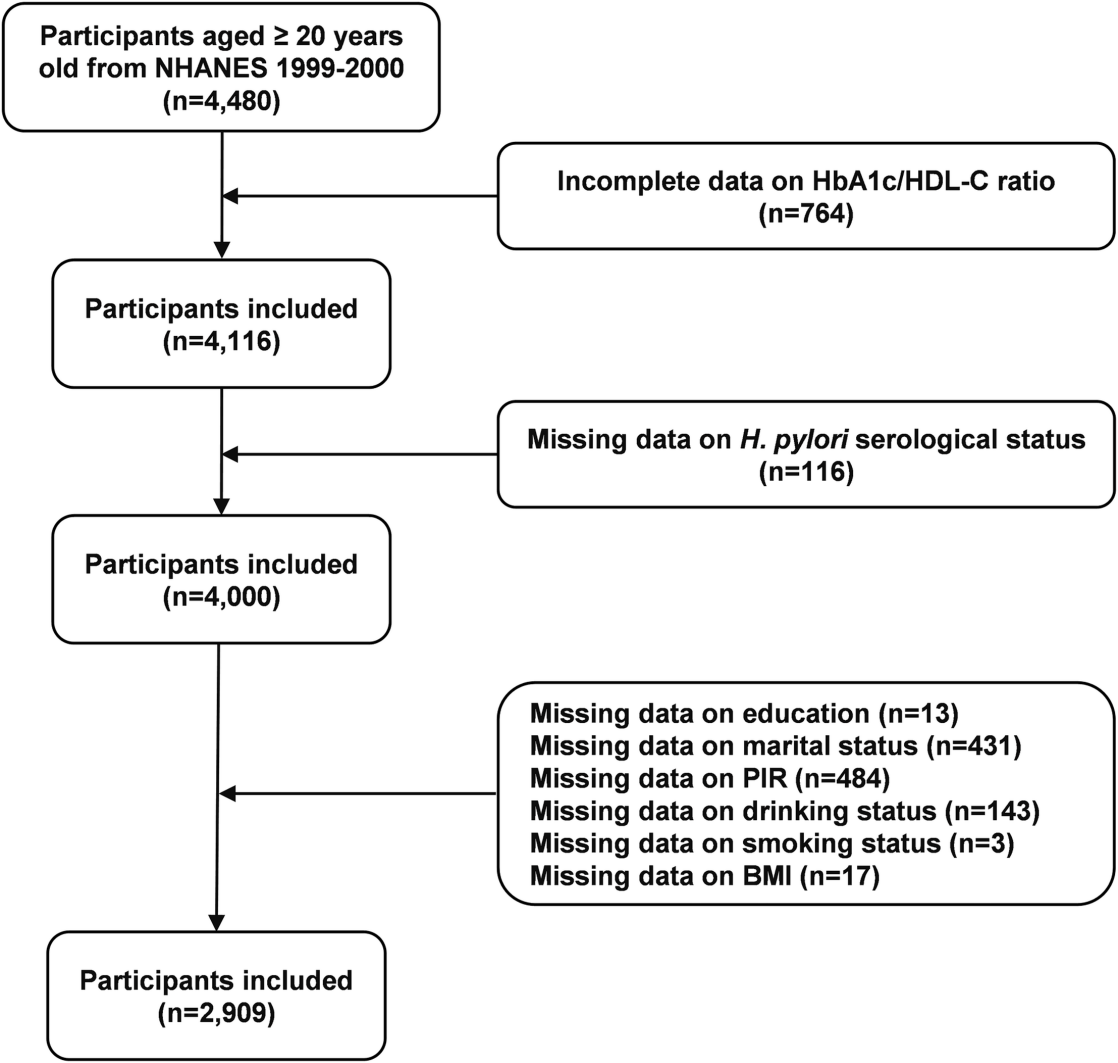
Flow chart of participants selection. *H. pylori*, *Helicobacter pylori*; PIR, poverty income ratio; BMI, body mass index; HbA1c, hemoglobin A1c; HDL-C, high-density lipoprotein cholesterol; NHANES, National Health and Nutrition Examination Survey.

This study utilized data from the 1999–2000 NHANES cycle. Initially, 4,480 participants aged 20 years or older were included. After excluding individuals with incomplete data on the HbA1c/HDL-C ratio (*n* = 764) and *H. pylori* serological status (*n* = 116), as well as those with missing covariates such as education level (*n* = 13), marital status (*n* = 431), poverty income ratio (PIR) (*n* = 484), drinking status (*n* = 143), smoking status (*n* = 3), and body mass index (BMI) (*n* = 17), a total of 2,909 participants were included in the final analysis (Figure 1).

### 2.2 Assessment of *Helicobacter pylori* seropositivity status

*Helicobacter pylori* seropositivity status was assessed using enzyme-linked immunosorbent assay (ELISA) to quantify anti-*H. pylori* immunoglobulin G (IgG) antibody levels (22). Participants were classified into two groups based on established ELISA cutoff values: *H. pylori-positive* (optical density [OD] value ≥1.1) and *H. pylori*-negative (OD value < 0.9). Ambiguous results within the range of 0.9 to 1.1 were excluded from the analysis to ensure precise statistical outcomes, consistent with previous epidemiological studies (23–25).

### 2.3 Assessment of the HbA1c/HDL-C ratio

Fasting blood samples were analyzed to measure HbA1c and HDL-C levels. The HbA1c/HDL-C ratio was calculated by dividing HbA1c (%) by HDL-C (mg/dL) (15). Participants were categorized into quartiles (Q1 to Q4) based on their HbA1c/HDL-C ratio values, with quartile thresholds determined by dividing the study population into four equal groups.

### 2.4 Assessment of covariates

To ensure comprehensive adjustments in the analysis, relevant sociodemographic characteristics, lifestyle behaviors, and comorbid health conditions were included as covariates. Sociodemographic variables encompassed age, sex, race, education level, marital status, and PIR. Lifestyle factors included drinking status (classified as <12 or ≥12 alcoholic drinks per year) and smoking status (categorized as never, former, or current smoker). Health conditions were assessed using a combination of self-reported data and objective clinical measurements. BMI was calculated as weight in kilograms divided by height in meters squared (kg/m^2^) and classified into normal weight (<25), overweight (≥25 and <30), and obesity (≥30) (26). Hypertension was defined as a self-reported physician diagnosis, use of antihypertensive medications, or elevated blood pressure (systolic ≥130 mmHg or diastolic ≥80 mmHg) (27). Cardiovascular disease, encompassing coronary heart disease, angina, congestive heart failure, myocardial infarction, and stroke, was ascertained through self-reported diagnoses provided by healthcare professionals (28).

### 2.5 Statistical analysis

All statistical analyses were conducted using appropriate NHANES sampling weights to account for the complex, multi-stage cluster survey design. Baseline characteristics of participants were described using weighted means (95% confidence intervals [CIs]) for continuous variables and weighted percentages (95% CIs) for categorical variables. Differences in baseline characteristics were assessed using weighted linear regression for continuous variables and weighted chi-square test for categorical variables.

The association between *H. pylori* seropositivity and the HbA1c/HDL-C ratio was evaluated using multivariable linear regression models. Crude model was unadjusted, Model 1 adjusted for age, sex, and race, and Model 2 further adjusted for education, marital status, PIR, drinking status, smoking status, BMI, hypertension, diabetes, and cardiovascular disease. Results were reported as beta (*β*) coefficients with corresponding 95% CIs. Subgroup analyses were conducted to examine the association between *H. pylori* seropositivity and the HbA1c/HDL-C ratio across different age, sex, race, PIR, BMI, and disease histories (hypertension and cardiovascular disease). Interaction tests were used to evaluate the consistency of these associations across subgroups.

As a sensitivity analysis, we performed logistic regression analyses with *H. pylori seropositivity* as the dependent variable and the HbA1c/HDL-C ratio as the independent variable. Both continuous and quartile-based forms of the HbA1c/HDL-C ratio were included in the regression analyses. Results were reported as odds ratios (ORs) with corresponding 95% CIs.

To explore potential non-linear relationships between *H. pylori* seropositivity and the HbA1c/HDL-C ratio, generalized additive models with smoothing splines were employed. Additionally, recursive algorithms and two-stage logistic models were utilized to detect any potential inflection points in the relationship. Likelihood ratio tests were performed to compare the fit of single logistic regression models with that of two-stage logistic models.

All analyses were performed using R (http://www.R-project.org, version 4.3.1) and EmpowerStats (http://www.empowerstats.com, version 4.2). Statistical significance was defined as a two-sided *p* value < 0.05.

## 3 Results

### 3.1 Baseline characteristics

This analysis included 2,909 U.S. adults with a weighted mean age of 44.65 years (95% CI: 43.59, 45.70), of whom 48.22% were male and 43.8% exhibited *H. pylori* seropositive. Table 1 summarizes the comparative analysis of demographic, socioeconomic, and clinical characteristics between *H. pylori* seropositive and seronegative participants. Within the *H. pylori* seropositive group, there was a greater proportion of older individuals and Mexican Americans. They also had lower levels of education, lower PIR, and were more likely to be married or living with a partner. Additionally, *H. pylori* seropositive participants were more likely to be smokers and less likely to consume alcohol. Furthermore, individuals with *H. pylori* seropositive exhibited higher prevalence rates of hypertension, diabetes, and cardiovascular disease. However, traditional metabolic markers, such as TC, TG, LDL-C, and uric acid, did not differ significantly between the two groups. Notably, the HbA1c/HDL-C ratio was significantly higher among *H. pylori* seropositive participants.

**Table 1:** Basic characteristics of the study population.

Characteristics	Overall (*n* = 2,909)	*H. pylori* seronegative (*n* = 1,655)	*H. pylori* seropositive (*n* = 1,254)	*p* value
Age (years)	44.65 (43.59, 45.70)	42.85 (41.55, 44.15)	48.95 (47.30, 50.60)	<0.001
Sex (%)				0.390
Male	48.22 (46.20, 50.25)	47.79 (45.47, 50.12)	49.26 (46.06, 52.47)	
Female	51.78 (49.75, 53.80)	52.21 (49.88, 54.53)	50.74 (47.53, 53.94)	
Race (%)				<0.001
Mexican American	5.91 (3.41, 10.05)	3.23 (1.86, 5.57)	12.33 (6.71, 21.56)	
Other Hispanic	8.02 (3.51, 17.29)	4.87 (2.13, 10.77)	15.58 (6.90, 31.46)	
Non-Hispanic White	72.86 (66.28, 78.58)	82.04 (77.07, 86.13)	50.83 (41.85, 59.76)	
Non-Hispanic Black	8.76 (5.62, 13.42)	6.32 (4.07, 9.69)	14.61 (8.85, 23.17)	
Other Race	4.45 (2.39, 8.15)	3.53 (1.70, 7.18)	6.65 (3.79, 11.41)	
Education (%)				<0.001
Less than high school	22.08 (19.30, 25.14)	14.42 (11.94, 17.31)	40.47 (37.08, 43.96)	
High school	25.97 (21.49, 31.03)	25.91 (20.16, 32.63)	26.13 (23.54, 28.90)	
More than high school	51.95 (46.20, 57.64)	59.67 (51.97, 66.92)	33.40 (30.05, 36.93)	
Marital status (%)				0.011
Never married	17.86 (15.23, 20.83)	19.54 (15.84, 23.85)	13.83 (10.66, 17.75)	
Widowed/divorced/separated	17.77 (15.01, 20.93)	16.58 (13.74, 19.88)	20.64 (17.43, 24.27)	
Married/living with partner	64.36 (59.71, 68.76)	63.88 (58.08, 69.30)	65.53 (60.86, 69.92)	
PIR (%)				<0.001
<1.3	23.24 (17.69, 29.89)	18.33 (12.58, 25.94)	35.00 (29.15, 41.35)	
1.3 to <3.5	34.72 (30.75, 38.92)	32.93 (28.32, 37.89)	39.03 (34.78, 43.45)	
≥3.5	42.04 (34.73, 49.72)	48.74 (39.81, 57.75)	25.97 (21.66, 30.79)	
Drinking (%)				0.045
<12 alcoholic drinks per year	27.14 (23.60, 30.98)	25.73 (22.40, 29.37)	30.52 (24.65, 37.10)	
≥12 alcoholic drinks per year	72.86 (69.02, 76.40)	74.27 (70.63, 77.60)	69.48 (62.90, 75.35)	
Smoking (%)				0.005
Never smoker	50.71 (46.38, 55.04)	53.20 (48.36, 57.97)	44.75 (39.09, 50.55)	
Former smoker	24.63 (22.06, 27.39)	24.29 (20.81, 28.14)	25.45 (21.53, 29.81)	
Current smoker	24.66 (21.23, 28.43)	22.52 (19.08, 26.38)	29.79 (24.75, 35.38)	
BMI (%)				0.764
<25	35.60 (31.76, 39.63)	35.84 (31.41, 40.53)	35.01 (30.24, 40.11)	
25–30	33.72 (30.55, 37.04)	33.93 (30.84, 37.15)	33.22 (28.26, 38.57)	
≥30	30.68 (27.81, 33.72)	30.23 (26.67, 34.05)	31.77 (29.41, 34.22)	
Hypertension (%)				0.001
No	50.37 (47.06, 53.68)	53.39 (49.13, 57.60)	43.11 (38.42, 47.93)	
Yes	49.63 (46.32, 52.94)	46.61 (42.40, 50.87)	56.89 (52.07, 61.58)	
Diabetes (%)				<0.001
No	92.10 (90.31, 93.58)	93.70 (92.24, 94.91)	88.25 (83.71, 91.65)	
Yes	7.90 (6.42, 9.69)	6.30 (5.09, 7.76)	11.75 (8.35, 16.29)	
Cardiovascular disease (%)				0.001
No	91.79 (90.29, 93.08)	92.90 (91.20, 94.29)	89.12 (86.50, 91.29)	
Yes	8.21 (6.92, 9.71)	7.10 (5.71, 8.80)	10.88 (8.71, 13.50)	
FBG (mg/dL)	100.38 (98.48, 102.29)	98.63 (96.30, 100.95)	105.01 (101.11, 108.91)	0.015
HbA1c (%)	5.40 (5.31, 5.48)	5.30 (5.22, 5.39)	5.62 (5.50, 5.74)	<0.001
TC (mg/dL)	202.58 (199.71, 205.44)	202.36 (199.08, 205.65)	203.09 (198.53, 207.64)	0.788
TG (mg/dL)	143.21 (134.63, 151.79)	139.42 (127.86, 150.98)	153.16 (138.68, 167.64)	0.204
HDL-C (mg/dL)	50.29 (48.95, 51.63)	51.10 (49.59, 52.60)	48.36 (46.54, 50.18)	0.018
LDL-C (mg/dL)	125.47 (122.28, 128.65)	125.26 (121.63, 128.88)	126.04 (122.37, 129.70)	0.700
Uric acid (mg/dL)	5.30 (5.21, 5.39)	5.27 (5.17, 5.37)	5.36 (5.21, 5.51)	0.313
HbA1c/HDL-C ratio	4.57 (4.39, 4.74)	4.42 (4.23, 4.61)	4.93 (4.71, 5.15)	<0.001

Data were presented as weighted means or percentages (95% CIs). *H. pylori*, *Helicobacter pylori*; PIR, poverty income ratio; BMI, body mass index; FBG, fasting blood glucose, HbA1c, hemoglobin A1c; HDL-C, high-density lipoprotein cholesterol; TC, total cholesterol; TG, triglycerides.

### 3.2 Association between *Helicobacter pylori* seropositivity and the HbA1c/HDL-C ratio

Table 2 presents the associations between *H. pylori* seropositivity and HbA1c/HDL-C ratio. The results indicated that *H. pylori* seropositivity was positively associated with the HbA1c/HDL-C ratio in the unadjusted model (*β*: 0.55, 95% CI: 0.41, 0.69), the partially adjusted model (*β*: 0.36, 95% CI: 0.22, 0.51), and the fully adjusted model (*β*: 0.28, 95% CI: 0.13, 0.42).

**Table 2:** Association of *H. pylori* seropositivity with the HbA1c/HDL-C ratio.

*H. pylori* seropositivity	Crude model		Model 1		Model 2	
	*β* (95% CI)	*p* value	*β* (95% CI)	*p* value	*β* (95% CI)	*p* value
Seropositive vs. Seronegative	0.55 (0.41, 0.69)	<0.001	0.36 (0.22, 0.51)	<0.001	0.28 (0.13, 0.42)	<0.001

*H. pylori*, *Helicobacter pylori*; HbA1c, hemoglobin A1c; HDL-C, high-density lipoprotein cholesterol.

Model 1: Adjusted for age, sex, and race.

Model 2: Adjusted for age, sex, race, education, marital status, PIR, drinking status, smoking status, BMI, hypertension, and cardiovascular disease.

### 3.3 Subgroup analysis

**Figure 2:**
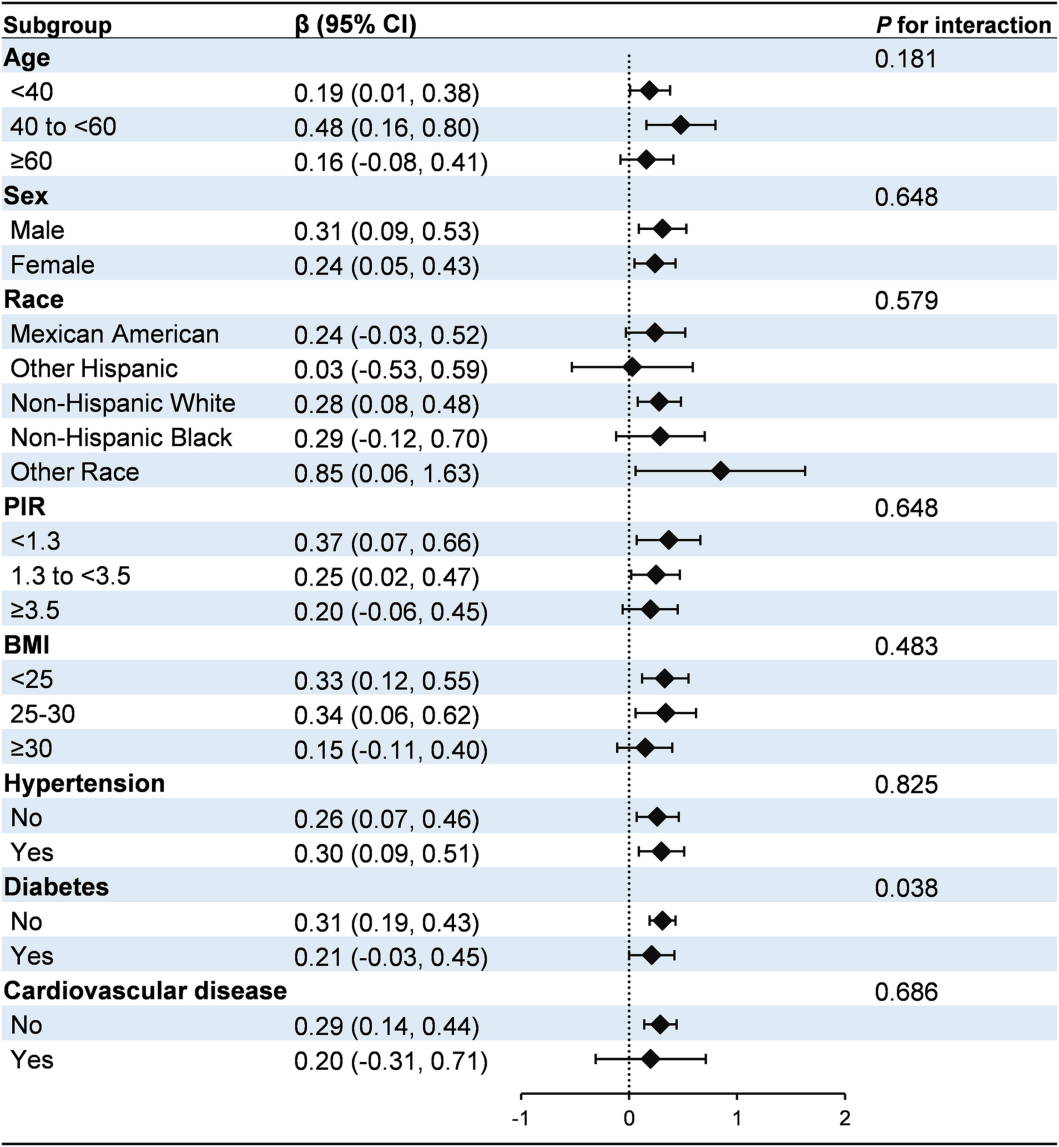
Subgroup analysis for the association between *H. pylori* seropositivity and the HbA1c/HDL-C ratio. The model was adjusted for age, sex, race, education, marital status, PIR, drinking, smoking, BMI, hypertension, diabetes, and cardiovascular disease. Diabetes was identified based on self-reported physician diagnosis, use of insulin or glucose-lowering medications, fasting blood glucose levels ≥126 mg/dL, or glycated hemoglobin levels ≥6.5%. PIR, poverty income ratio; BMI, body mass index.

Subgroup analyses and interaction tests were conducted to evaluate the consistency of the association between *H. pylori* seropositivity and the HbA1c/HDL-C ratio across various demographic and clinical subgroups, including age, sex, race, PIR, BMI, hypertension, diabetes, and cardiovascular disease (Figure 2). The positive association between *H. pylori* seropositivity and the HbA1c/HDL-C ratio was generally consistent across most subgroups (*P* for interaction >0.05). However, a significant interaction was identified for diabetes status (*P* for interaction = 0.038). Specifically, the association was stronger in individuals without diabetes (*β*: 0.31, 95% CI: 0.19, 0.43) compared to those with diabetes (*β*: 0.21, 95% CI: −0.03, 0.45).

### 3.4 Sensitivity analysis

To further validate the robustness of our findings, we conducted additional sensitivity analyses. In the fully adjusted model, participants in the highest quartile (Q4) of the HbA1c/HDL-C ratio exhibited an 84% increased probability of *H. pylori* seropositivity (OR: 1.75, 95% CI: 1.38, 2.44) compared to those in the lowest quartile (Q1). Notably, trend tests confirmed the statistical significance of the overall positive association between the HbA1c/HDL-C ratio and *H. pylori* seropositivity (all *P* for trend <0.05) (Supplementary Table 1).

### 3.5 Dose–response relationship between *Helicobacter pylori* seropositivity and the HbA1c/HDL-C ratio

**Figure 3:**
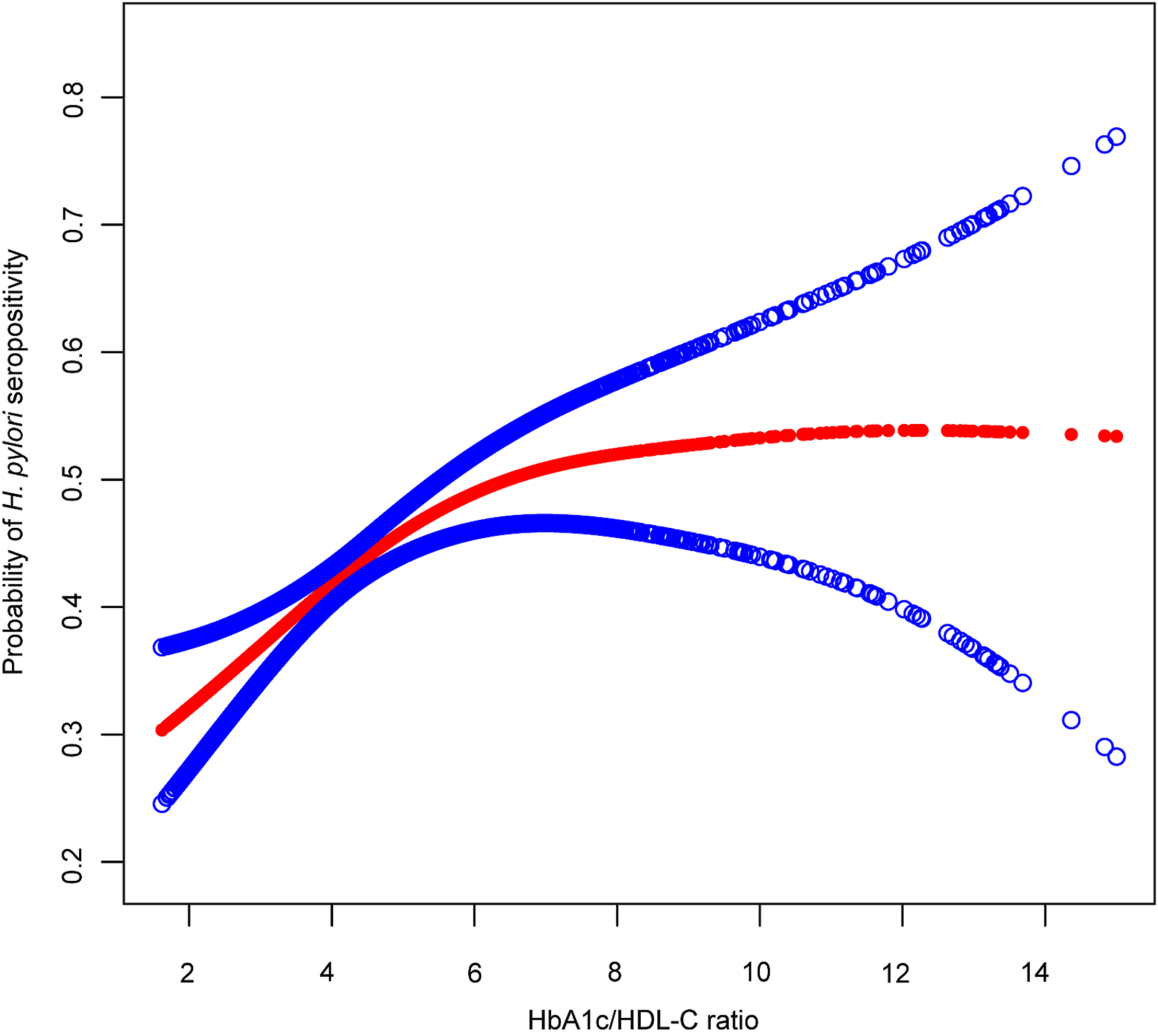
The association between the HbA1c/HDL-C ratio and *H. pylori* seropositivity. The red line represents the smooth curve fit between variables, with blue bands representing the 95% CI of the fit. The model was adjusted for age, sex, race, education, marital status, PIR, drinking, smoking, BMI, hypertension, and cardiovascular disease. *H. pylori*, *Helicobacter pylori*; HbA1c, hemoglobin A1c; HDL-C, high-density lipoprotein cholesterol.

Generalized additive models with smoothing splines revealed a nonlinear, L-shaped association between the HbA1c/HDL-C ratio and *H. pylori* seropositivity (Figure 3). Threshold effect analysis identified an inflection point at an HbA1c/HDL-C ratio of 4.81. Below this threshold, a significant positive association was observed between the HbA1c/HDL-C ratio and *H. pylori* seropositivity (OR: 1.29, 95% CI: 1.14, 1.46), whereas no significant association was observed above the threshold (OR: 1.02, 95% CI: 0.96, 1.09). The log-likelihood ratio test confirmed the superiority of the two-piecewise linear regression model over the one-line linear model (*p* = 0.003) (Supplementary Table 2). We constructed stratified curves for the association between the HbA1c/HDL-C ratio and *H. pylori* seropositivity by diabetes status (Supplementary Figure 1). In non-diabetic individuals, the HbA1c/HDL-C ratio exhibited a linear association with *H. pylori* seropositivity (OR: 1.19, 95% CI: 1.10, 1.28), and the log-likelihood ratio test (*p* = 0.205) supported the adequacy of the linear model. Among diabetic individuals, no significant association between the HbA1c/HDL-C ratio and *H. pylori seropositivity* was found (Supplementary Table 3).

## 4 Discussion

In this cross-sectional study conducted among U.S. population, we identified a significant positive association between *H. pylori* seropositivity and the HbA1c/HDL-C ratio. Notably, we observed a “L”-shaped relationship between *H. pylori* seropositivity and the HbA1c/HDL-C ratio, with an inflection point at 4.81. Below this threshold, *H. pylori* seropositivity was positively associated with the HbA1c/HDL-C ratio. However, above this value, no significant association was detected.

*H. pylori* infection may increase the risk of metabolic dysfunction. A systematic review has reported a positive correlation between *H. pylori* infection and metabolic syndrome, with infected individuals exhibiting elevated levels of TG, fasting blood glucose (FBG), BMI, and reduced HDL-C (29). Several studies have also demonstrated that *H. pylori* infection is linked to abnormal glucose metabolism and an increased risk of diabetes (30–32). Specifically, individuals with *H. pylori* infection exhibit significantly higher HbA1c levels compared to uninfected individuals, and a positive correlation has been observed between *H. pylori* infection and elevated HbA1c levels in diabetic patients (33). These findings suggest that *H. pylori* infection may be closely associated with IR-related disorders. However, the relationship between *H. pylori* infection and markers such as HbA1c and IR remains controversial. For instance, a large cross-sectional study involving 37,263 participants found that *H. pylori* infection was associated only with dyslipidemia, showing no significant correlation with FBG or HbA1c levels (34). In addition to its potential impact on glucose metabolism, *H. pylori* infection has been shown to significantly influence lipid profiles. Studies indicate that individuals with *H. pylori* infection tend to exhibit elevated levels of TC, TG and LDL-C, alongside reduced HDL-C levels (35–37). Notably, the prevalence of dyslipidemia is significantly lower in individuals who have undergone *H. pylori* eradication compared to those with active infection (38). Eradication of *H. pylori* has been associated with improved lipid profiles, including increased HDL-C levels and decreased TG and LDL-C levels (39, 40). These findings demonstrate the complexity of the relationship between *H. pylori* infection and metabolic disorders and suggest that more research is needed to focus on the association between *H. pylori* infection and abnormal glucose and lipid metabolism.

Glucose and lipid metabolism interact through complex physiological and pathological processes (41, 42). The triglyceride-glucose (TyG) index, derived from FPG and TG levels, has emerged as a reliable marker of IR and metabolic dysfunction (43, 44). Previous studies have shown that an elevated TyG index is associated with an increased risk of *H. pylori* infection (24, 45). Compared to the short-term glucose indicators used in the TyG index, HbA1c reflects long-term glucose control and provides a more stable measure than fasting glucose (46). Our findings are consistent with studies using the TyG index, revealing the close association between *H. pylori* seropositivity and glycolipid metabolism. The HbA1c/HDL-C ratio may offer more stable and clinically meaningful insights into the relationship between *H. pylori* infection and metabolic dysfunction.

In this study, we identified a saturation effect in the relationship between *H. pylori* seropositivity and the HbA1c/HDL-C ratio using segmented regression analysis. Within the lower range of the HbA1c/HDL-C ratio, mild disturbances in glucose and lipid metabolism were associated with a higher probability of *H. pylori* seropositivity. However, beyond a certain threshold, this association was no longer significant. This saturation effect aligns with previous observations of nonlinear relationships between metabolic indicators and health outcomes (16, 47), suggesting that the association between metabolic status and *H. pylori* infection may vary depending on the severity of metabolic disturbances.

Subgroup analyses revealed that the association between *H. pylori* seropositivity and the HbA1c/HDL-C ratio was more pronounced in individuals without diabetes than in those with diabetes. This discrepancy may be attributed to the complex metabolic disturbances in individuals with diabetes, characterized by hyperglycemia and dyslipidemia, which could obscure the relationship between *H. pylori* seropositivity and the HbA1c/HDL-C ratio (48, 49). Unfortunately, our study lacked detailed data on disease duration, treatment regimens, and the presence of complications, limiting our ability to explore these potential influences. Additionally, glycemic and lipid-lowering therapies, which are common in diabetes management, may alter the measurement of the HbA1c/HDL-C ratio and impact *H. pylori* colonization or eradication, thereby attenuating the observed association (50, 51). Our findings suggest that *H. pylori* seropositivity is positively associated with the HbA1c/HDL-C ratio, especially in non-diabetic populations. Future research should explore the mechanisms underlying this association and develop personalized strategies for metabolic management.

The potential mechanisms underlying the association between the HbA1c/HDL-C ratio and *H. pylori* infection are multifaceted. *H. pylori* infection induces systemic inflammation through the release of pro-inflammatory cytokines such as TNF-*α*, IL-1, and IL-6, which disrupt insulin signaling pathways and exacerbate insulin resistance (52, 53). Moreover, *H. pylori* infection may alter the gastric environment, influencing gut microbiota composition and contributing to systemic inflammation and metabolic dysregulation (54, 55). The relationship between metabolic disorders and *H. pylori* infection appears to be bidirectional. Metabolic dysfunction, characterized by IR, hyperglycemia, and dyslipidemia, promotes oxidative stress and impairs gastric mucosal defenses, thereby creating a favorable environment for *H. pylori* colonization and persistence (10, 56, 57). Additionally, IR-related autonomic dysfunction may slow gastrointestinal motility and reduce gastric acid secretion, further facilitating *H. pylori* survival (58–60). These interconnected mechanisms highlight the complex interplay between metabolic health and *H. pylori* infection, with the exact mechanisms warranting further investigation.

This study is the first to investigate the association between *H. pylori* seropositivity and the HbA1c/HDL-C ratio. A key strength of this study is its use of a large, nationally representative sample of U.S. adults, which enhances the generalizability of the findings to broader populations. However, several limitations should be noted. First, as a cross-sectional study, it cannot establish a causal relationship between *H. pylori* seropositivity and the HbA1c/HDL-C ratio. Further longitudinal studies are needed to clarify the directionality of this association. Second, the NHANES database provides only serological data on *H. pylori* infection status, which precludes differentiation between past and current infections among seropositive participants. Although serology is a widely used and reliable method for assessing *H. pylori* status in large epidemiological studies (25, 28), it does not capture the activity of infection, thereby limiting our ability to assess the relationship between chronic versus acute infection and metabolic disturbances. Third, there may be unmeasured confounding factors, such as genetic predisposition, unaccounted medication use, and environmental influences, which could affect the observed association between *H. pylori* seropositivity and the HbA1c/HDL-C ratio. Finally, the study focused exclusively on a U.S. population, which may limit the generalizability of the findings to other ethnic or geographic groups.

## 5 Conclusion

This study identifies a positive association between *H. pylori* seropositivity and the HbA1c/HDL-C ratio in a nationally representative sample. The findings suggest that this metabolic marker may serve as a potential indicator of *H. pylori* infection in clinical settings. Future longitudinal studies are needed to establish causality and explore underlying mechanisms.

## Data Availability

The original contributions presented in the study are included in the article/Supplementary material, further inquiries can be directed to the corresponding author.

## References

[1] Li Y Choi H Leung K Jiang F Graham DY Leung WK. Global prevalence of *Helicobacter pylori* infection between 1980 and 2022: a systematic review and meta-analysis. Lancet Gastroenterol Hepatol. (2023) 8:553–64. doi: 10.1016/S2468-1253(23)00070-5, PMID: 37086739

[2] Chen YC Malfertheiner P Yu HT Kuo CL Chang YY Meng FT. Global prevalence of *Helicobacter pylori* infection and incidence of gastric Cancer between 1980 and 2022. Gastroenterology. (2024) 166:605–19. doi: 10.1053/j.gastro.2023.12.022, PMID: 38176660

[3] Malfertheiner P Camargo MC El-Omar E Liou JM Peek R Schulz C. *Helicobacter pylori* infection. Nat Rev Dis Primers. (2023) 9:19. doi: 10.1038/s41572-023-00431-8, PMID: 37081005 PMC11558793

[4] Usui Y Taniyama Y Endo M Koyanagi YN Kasugai Y Oze I. *Helicobacter pylori*, homologous-recombination genes, and gastric Cancer. N Engl J Med. (2023) 388:1181–90. doi: 10.1056/NEJMoa2211807, PMID: 36988593

[5] Kountouras J Papaefthymiou A Polyzos SA Liatsos C Tzitiridou-Chatzopoulou M Chatzopoulos D. Potential impact of Helicobacter pylori and metabolic syndrome-related non-alcoholic fatty liver disease on cardio-cerebrovascular disease. Metabolism. (2022) 135:155276. doi: 10.1016/j.metabol.2022.155276, PMID: 35940250

[6] Sun L Zheng H Qiu M Hao S Liu X Zhu X. *Helicobacter pylori* infection and risk of cardiovascular disease. Helicobacter. (2023) 28:e12967. doi: 10.1111/hel.12967, PMID: 36974892

[7] Wang F Yao Z Jin T Mao B Shao S Shao C. Research progress on *Helicobacter pylori* infection related neurological diseases. Ageing Res Rev. (2024) 99:102399. doi: 10.1016/j.arr.2024.102399, PMID: 38955263

[8] Chen C Zhang C Wang X Zhang F Zhang Z Ma P. *Helicobacter pylori* infection may increase the severity of nonalcoholic fatty liver disease via promoting liver function damage, glycometabolism, lipid metabolism, inflammatory reaction and metabolic syndrome. Eur J Gastroenterol Hepatol. (2020) 32:857–66. doi: 10.1097/MEG.0000000000001601, PMID: 31714387 PMC7269023

[9] Izhari MA Al Mutawa OA Mahzari A Alotaibi EA Almashary MA Alshahrani JA. *Helicobacter pylori* (*H. pylori*) infection-associated dyslipidemia in the Asir region of Saudi Arabia. Life (Basel). (2023) 13:2206. doi: 10.3390/life13112206, PMID: 38004346 PMC10672336

[10] Qiu J Yu Y Liu D Chen S Wang Y Peng J. Association between non-insulin-based insulin resistance surrogate makers and *Helicobacter pylori* infection: a population-based study. BMC Gastroenterol. (2025) 25:25. doi: 10.1186/s12876-025-03610-x, PMID: 39838324 PMC11753134

[11] Baradaran A Dehghanbanadaki H Naderpour S Pirkashani LM Rajabi A Rashti R. The association between Helicobacter pylori and obesity: a systematic review and meta-analysis of case-control studies. Clin Diabetes Endocrinol. (2021) 7:15. doi: 10.1186/s40842-021-00131-w, PMID: 34243821 PMC8272347

[12] Balintescu A Mårtensson J. Hemoglobin A1c and permissive hyperglycemia in patients in the intensive care unit with diabetes. Crit Care Clin. (2019) 35:289–300. doi: 10.1016/j.ccc.2018.11.010, PMID: 30784610

[13] Yazdanpanah S Rabiee M Tahriri M Abdolrahim M Rajab A Jazayeri HE. Evaluation of glycated albumin (GA) and GA/HbA1c ratio for diagnosis of diabetes and glycemic control: a comprehensive review. Crit Rev Clin Lab Sci. (2017) 54:219–32. doi: 10.1080/10408363.2017.1299684, PMID: 28393586

[14] Mineo C Shaul PW. Novel biological functions of high-density lipoprotein cholesterol. Circ Res. (2012) 111:1079–90. doi: 10.1161/CIRCRESAHA.111.258673, PMID: 23023510 PMC3500606

[15] Hu X Li W Wang C Zhang H Lu H Li G. Association between the plasma-glycosylated hemoglobin A1c/high-density lipoprotein cholesterol ratio and carotid atherosclerosis: a retrospective study. J Diabetes Res. (2021) 2021:1–10. doi: 10.1155/2021/9238566, PMID: 34805413 PMC8598339

[16] Huang C You H Zhang Y Fan L Feng X Shao N. Association between the hemoglobin A1c/high-density lipoprotein cholesterol ratio and stroke incidence: a prospective nationwide cohort study in China. Lipids Health Dis. (2025) 24:25. doi: 10.1186/s12944-025-02438-4, PMID: 39863906 PMC11762894

[17] He S Lu S Yu C Kuang M Qiu J Sheng G. The newly proposed plasma-glycosylated hemoglobin A1c/high-density lipoprotein cholesterol ratio serves as a simple and practical indicator for screening metabolic associated fatty liver disease: an observational study based on a physical examination population. BMC Gastroenterol. (2024) 24:274. doi: 10.1186/s12876-024-03362-0, PMID: 39160462 PMC11331873

[18] Ogden CL Carroll MD Kit BK Flegal KM. Prevalence of obesity and trends in body mass index among US children and adolescents, 1999-2010. JAMA. (2012) 307:483–90. doi: 10.1001/jama.2012.40, PMID: 22253364 PMC6362452

[19] XuCSongZWangJNLiCC. Association of visceral adiposity index with phenotypic age acceleration: insight from NHANES 1999-2010. J Nutr Health Aging. (2024) 28:100323. doi: 10.1016/j.jnha.2024.100323, PMID: 39067143

[20] Paulose-Ram R Graber JE Woodwell D Ahluwalia N. The National Health and nutrition examination survey (NHANES), 2021-2022: adapting data collection in a COVID-19 environment. Am J Public Health. (2021) 111:2149–56. doi: 10.2105/AJPH.2021.306517, PMID: 34878854 PMC8667826

[21] Song Z Gu HQ Xu C. Association of the non-high-density lipoprotein cholesterol to high-density lipoprotein cholesterol ratio with non-alcoholic fatty liver disease and hepatic steatosis in United States adults: insights from NHANES 2017-2020. Front Nutr. (2025) 12:1540903. doi: 10.3389/fnut.2025.1540903, PMID: 40290661 PMC12021641

[22] Berrett AN Gale SD Erickson LD Brown BL Hedges DW. Folate and inflammatory markers moderate the association between *Helicobacter pylori* exposure and cognitive function in US adults. Helicobacter. (2016) 21:471–80. doi: 10.1111/hel.12303, PMID: 26935014

[23] Xiong YJ Du LL Diao YL Wen J Meng XB Gao J. Association of dietary inflammatory index with *helicobacter pylori* infection and mortality among US population. J Transl Med. (2023) 21:538. doi: 10.1186/s12967-023-04398-8, PMID: 37573314 PMC10422799

[24] Zhu XY Xiong YJ Meng XD Xu HZ Huo L Deng W. Association of triglyceride-glucose index with *helicobacter pylori* infection and mortality among the US population. Diabetol Metab Syndr. (2024) 16:187. doi: 10.1186/s13098-024-01422-9, PMID: 39090745 PMC11293276

[25] Qiu J Fang H Liu D Lai Q Xie J Wang Y. Accelerated biological aging mediates the association between inflammatory markers with *Helicobacter pylori* infection and mortality. J Transl Med. (2025) 23:174. doi: 10.1186/s12967-025-06189-9, PMID: 39930506 PMC11812229

[26] Chen C Ye Y Zhang Y Pan XF Pan A. Weight change across adulthood in relation to all cause and cause specific mortality: prospective cohort study. BMJ. (2019) 367:l5584. doi: 10.1136/bmj.l5584, PMID: 31619383 PMC6812615

[27] Whelton PK Carey RM Aronow WS Casey DE Jr Collins KJ Dennison C. 2017 ACC/AHA/AAPA/ABC/ACPM/AGS/APhA/ASH/ASPC/NMA/PCNA guideline for the prevention, detection, evaluation, and management of high blood pressure in adults: a report of the American College of Cardiology/American Heart Association task force on clinical practice guidelines. J Am Coll Cardiol. (2018) 71:e127–248. doi: 10.1016/j.jacc.2017.11.006 29146535

[28] Tang C Zhang Q Zhang C Du X Zhao Z Qi W. Relationships among *Helicobacter pylori* seropositivity, the triglyceride-glucose index, and cardiovascular disease: a cohort study using the NHANES database. Cardiovasc Diabetol. (2024) 23:441. doi: 10.1186/s12933-024-02536-0, PMID: 39695657 PMC11657082

[29] Upala S Jaruvongvanich V Riangwiwat T Jaruvongvanich S Sanguankeo A. Association between *Helicobacter pylori* infection and metabolic syndrome: a systematic review and meta-analysis. J Dig Dis. (2016) 17:433–40. doi: 10.1111/1751-2980.12367, PMID: 27273478

[30] Hsieh MC Wang SS Hsieh YT Kuo FC Soon MS Wu DC. *Helicobacter pylori* infection associated with high HbA1c and type 2 diabetes. Eur J Clin Investig. (2013) 43:949–56. doi: 10.1111/eci.12124, PMID: 23879740

[31] Wan Z Song L Hu L Hu M Lei X Huang Y. *Helicobacter pylori* infection is associated with diabetes among Chinese adults. J Diabetes Investig. (2020) 11:199–205. doi: 10.1111/jdi.13102, PMID: 31207188 PMC6944826

[32] Mansori K Moradi Y Naderpour S Rashti R Moghaddam AB Saed L. *Helicobacter pylori* infection as a risk factor for diabetes: a meta-analysis of case-control studies. BMC Gastroenterol. (2020) 20:77. doi: 10.1186/s12876-020-01223-0, PMID: 32209055 PMC7092473

[33] Chen J Xing Y Zhao L Ma H. The association between *Helicobacter pylori* infection and glycated hemoglobin a in diabetes: a Meta-analysis. J Diabetes Res. (2019) 2019:1–10. doi: 10.1155/2019/3705264, PMID: 31583248 PMC6754895

[34] Kim TJ Lee H Kang M Kim JE Choi YH Min YW. *Helicobacter pylori* is associated with dyslipidemia but not with other risk factors of cardiovascular disease. Sci Rep. (2016) 6:38015. doi: 10.1038/srep38015, PMID: 27892538 PMC5125092

[35] Shimamoto T Yamamichi N Gondo K Takahashi Y Takeuchi C Wada R. The association of *Helicobacter pylori* infection with serum lipid profiles: An evaluation based on a combination of meta-analysis and a propensity score-based observational approach. PLoS One. (2020) 15:e0234433. doi: 10.1371/journal.pone.0234433, PMID: 32511269 PMC7279579

[36] Tali LDN Faujo GFN Konang JLN Dzoyem JP Kouitcheu LBM. Relationship between active *Helicobacter pylori* infection and risk factors of cardiovascular diseases, a cross-sectional hospital-based study in a sub-Saharan setting. BMC Infect Dis. (2022) 22:731. doi: 10.1186/s12879-022-07718-3, PMID: 36096730 PMC9469600

[37] Nigatie M Melak T Asmelash D Worede A. Dyslipidemia and its associated factors among *Helicobacter pylori*-infected patients attending at University of Gondar Comprehensive Specialized Hospital, Gondar, north-West Ethiopia: a comparative cross-sectional study. J Multidiscip Healthc. (2022) 15:1481–91. doi: 10.2147/JMDH.S368832, PMID: 35873092 PMC9297042

[38] Park Y Kim TJ Lee H Yoo H Sohn I Min YW. Eradication of *Helicobacter pylori* infection decreases risk for dyslipidemia: a cohort study. Helicobacter. (2021) 26:e12783. doi: 10.1111/hel.12783, PMID: 33508177

[39] Iwai N Okuda T Oka K Hara T Inada Y Tsuji T. *Helicobacter pylori* eradication increases the serum high density lipoprotein cholesterol level in the infected patients with chronic gastritis: a single-center observational study. PLoS One. (2019) 14:e0221349. doi: 10.1371/journal.pone.0221349, PMID: 31419266 PMC6697333

[40] Mokhtare M Mirfakhraee H Arshad M Samadani Fard SH Bahardoust M Movahed A. The effects of *helicobacter pylori* eradication on modification of metabolic syndrome parameters in patients with functional dyspepsia. Diabetes Metab Syndr. (2017) 11:S1031–5. doi: 10.1016/j.dsx.2017.07.035, PMID: 28780229

[41] Agbu P Carthew RW. MicroRNA-mediated regulation of glucose and lipid metabolism. Nat Rev Mol Cell Biol. (2021) 22:425–38. doi: 10.1038/s41580-021-00354-w, PMID: 33772227 PMC8853826

[42] Wat LW Svensson KJ. Novel secreted regulators of glucose and lipid metabolism in the development of metabolic diseases. Diabetologia. (2024) 67:2626–36. doi: 10.1007/s00125-024-06253-x, PMID: 39180580 PMC12087937

[43] Dang K Wang X Hu J Zhang Y Cheng L Qi X. The association between triglyceride-glucose index and its combination with obesity indicators and cardiovascular disease: NHANES 2003-2018. Cardiovasc Diabetol. (2024) 23:8. doi: 10.1186/s12933-023-02115-9, PMID: 38184598 PMC10771672

[44] Yao Y Wang B Geng T Chen J Chen W Li L. The association between TyG and all-cause/non-cardiovascular mortality in general patients with type 2 diabetes mellitus is modified by age: results from the cohort study of NHANES 1999-2018. Cardiovasc Diabetol. (2024) 23:43. doi: 10.1186/s12933-024-02120-6, PMID: 38281973 PMC10823741

[45] Liu W An J Jiao C Guo J Zhang L Jin H. Association of triglyceride-glucose index with *Helicobacter pylori* infection in the 1999-2000 NHANES cross-sectional study. Sci Rep. (2025) 15:387. doi: 10.1038/s41598-024-84536-4, PMID: 39747541 PMC11695683

[46] American Diabetes Association. 2. Classification and diagnosis of diabetes: standards of medical care in diabetes—2021. Diabetes Care. (2021) 44:S15–33. doi: 10.2337/dc21-S002 33298413

[47] Chen Y Yang C You N Zhang J. Relationship between Helicobacter pylori and glycated hemoglobin: a cohort study. Front Cell Infect Microbiol. (2023) 13:1196338. doi: 10.3389/fcimb.2023.1196338, PMID: 37360526 PMC10288807

[48] Kløve S Stinson SE Romme FO Butt J Graversen KB Lund MAV. *Helicobacter pylori* seropositivity associates with hyperglycemia, but not obesity, in Danish children and adolescents. BMC Med. (2024) 22:379. doi: 10.1186/s12916-024-03591-w, PMID: 39256870 PMC11389555

[49] Yang C You N Chen Y Zhang J. *Helicobacter pylori* infection increases the risk of dyslipidemia in Chinese diabetic population: a retrospective cross-sectional study. BMC Infect Dis. (2024) 24:730. doi: 10.1186/s12879-024-09597-2, PMID: 39054452 PMC11270938

[50] Courtois S Bénéjat L Izotte J Mégraud F Varon C Lehours P. Metformin can inhibit *Helicobacter pylori* growth. Future Microbiol. (2018) 13:1575–83. doi: 10.2217/fmb-2018-0184, PMID: 30421627

[51] Ness A Levi Z Belfer RG Dickman R Boltin D. Improvement in *Helicobacter pylori* eradication among adults receiving Semaglutide: a population-based propensity-score-adjusted analysis. Helicobacter. (2025) 30:e70014. doi: 10.1111/hel.70014, PMID: 39902748 PMC11792433

[52] Tang L Tang B Lei Y Yang M Wang S Hu S. *Helicobacter pylori*-induced Heparanase promotes *H. pylori* colonization and gastritis. Front Immunol. (2021) 12:675747. doi: 10.3389/fimmu.2021.675747, PMID: 34220822 PMC8248549

[53] Zhou X Liu W Gu M Zhou H Zhang G. *Helicobacter pylori* infection causes hepatic insulin resistance by the c-Jun/miR-203/SOCS3 signaling pathway. J Gastroenterol. (2015) 50:1027–40. doi: 10.1007/s00535-015-1051-6, PMID: 25689935

[54] Chen CC Liou JM Lee YC Hong TC El-Omar EM Wu MS. The interplay between Helicobacter pylori and gastrointestinal microbiota. Gut Microbes. (2021) 13:1–22. doi: 10.1080/19490976.2021.1909459, PMID: 33938378 PMC8096336

[55] Nabavi-Rad A Sadeghi A Asadzadeh Aghdaei H Yadegar A Smith SM Zali MR. The double-edged sword of probiotic supplementation on gut microbiota structure in *Helicobacter pylori* management. Gut Microbes. (2022) 14:2108655. doi: 10.1080/19490976.2022.2108655, PMID: 35951774 PMC9373750

[56] Aslan M Horoz M Nazligul Y Bolukbas C Bolukbas FF Selek S. Insulin resistance in H pylori infection and its association with oxidative stress. World J Gastroenterol. (2006) 12:6865–8. doi: 10.3748/wjg.v12.i42.6865, PMID: 17106938 PMC4087444

[57] Sheu SM Cheng H Kao CY Yang YJ Wu JJ Sheu BS. Higher glucose level can enhance the *H. pylori* adhesion and virulence related with type IV secretion system in AGS cells. J Biomed Sci. (2014) 21:96. doi: 10.1186/s12929-014-0096-9, PMID: 25296847 PMC4196111

[58] Kountouras J Boziki M Kazakos E Theotokis P Kesidou E Nella M. Impact of Helicobacter pylori and metabolic syndrome on mast cell activation-related pathophysiology and neurodegeneration. Neurochem Int. (2024) 175:105724. doi: 10.1016/j.neuint.2024.105724, PMID: 38508416

[59] Abdalla MMI. Enteric neuropathy in diabetes: implications for gastrointestinal function. World J Gastroenterol. (2024) 30:2852–65. doi: 10.3748/wjg.v30.i22.2852, PMID: 38947292 PMC11212710

[60] Chen J Chen L Sanseau P Freudenberg JM Rajpal DK. Significant obesity-associated gene expression changes occur in the stomach but not intestines in obese mice. Physiol Rep. (2016) 4:e12793. doi: 10.14814/phy2.12793, PMID: 27207783 PMC4886165

