# Supplementary material for: Association between Helicobacter pylori seropositivity and the hemoglobin A1c/high-density lipoprotein cholesterol ratio in U.S. adults: evidence from NHANES
Source: Front Nutr. 2025 Jun 9;12:1589510. doi: 10.3389/fnut.2025.1589510 (PMC12183076; doi:10.3389/fnut.2025.1589510)
Supplement: Supplementary file 1 [file Data_Sheet_1.pdf]

Association between *Helicobacter pylori* seropositivity and the hemoglobin A1c/high-density lipoprotein cholesterol ratio in U.S. adults: evidence from NHANES.

Supplementary Material

Supplementary Table 1 Sensitivity analysis of the association between the HbA1c/HDL-C ratio and *H. pylori* seropositivity

Supplementary Table 2 Threshold effect analysis of association between the HbA1c/HDL-C ratio and *H. pylori* seropositivity

Supplementary Table 3 Threshold effect analysis of association between the HbA1c/HDL-C ratio and *H. pylori* seropositivity stratified by diabetes status

Supplementary Figure 1 The association between the HbA1c/HDL-C ratio and *H. pylori* seropositivity stratified by diabetes status

Supplementary Table 1 Sensitivity analysis of the association between the HbA1c/HDL-C ratio and *H. pylori* seropositivity

	Crude Model		Model 1		Model 2	
	OR (95% CI)	*P* value	OR (95% CI)	*P* value	OR (95% CI)	*P* value
Continuous	1.17 (1.12, 1.22)	<0.001	1.11 (1.06, 1.17)	<0.001	1.09 (1.04, 1.15)	<0.001
Categories						
Q1	Reference		Reference		Reference	
Q2	1.30 (1.05, 1.61)	0.016	1.20 (0.95, 1.52)	0.132	1.26 (0.99, 1.62)	0.064
Q3	1.63 (1.32, 2.02)	<0.001	1.49 (1.17, 1.88)	0.001	1.49 (1.15, 1.91)	0.002
Q4	2.20 (1.78, 2.71)	<0.001	1.80 (1.41, 2.30)	<0.001	1.75 (1.38, 2.44)	<0.001
P for trend		<0.001		<0.001		<0.001

Model 1: Adjusted for age, sex, and race.

Model 2: Adjusted for age, sex, race, education, marital status, PIR, drinking status, smoking status, BMI, hypertension, and cardiovascular disease.

Abbreviations: *H. pylori*, *Helicobacter pylori*; HbA1c, hemoglobin A1c; HDL-C, high-density lipoprotein cholesterol; Q, quartile.

Supplementary Table 2 Threshold effect analysis of association between the HbA1c/HDL-C ratio and *H. pylori* seropositivity

	OR (95% CI)	*P* value
One - line logistic regression model	1.09 (1.04, 1.15)	<0.001
Two - piecewise logistic regression model		
HbA1c/HDL-C ≤ 4.81	1.29 (1.14, 1.46)	<0.001
HbA1c/HDL-C >4.81	1.02 (0.96, 1.09)	0.496
Log - likelihood ratio test		0.003

Abbreviations: *H. pylori*, *Helicobacter pylori*; HbA1c, hemoglobin A1c; HDL-C, high-density lipoprotein cholesterol.

Supplementary Table 3 Threshold effect analysis of association between the HbA1c/HDL-C ratio and *H. pylori* seropositivity stratified by diabetes status

	Non-Diabetes	Diabetes
One - line logistic regression model	1.19 (1.10, 1.28) ***	1.02 (0.93, 1.12)
Two - piecewise logistic regression model		
Inflection point (K)	4.27	4.15
HbA1c/HDL-C≤ K	1.31 (1.10, 1.55) **	2.06 (0.82, 5.16)
HbA1c/HDL-C >K	1.12 (1.01, 1.25) *	1.00 (0.90, 1.10)
Log-likelihood ratio test	0.205	0.124

***P<0.001; **P<0.01; *P<0.05

Diabetes was identified based on self-reported physician diagnosis, use of insulin or glucose-lowering medications, fasting blood glucose levels ≥126 mg/dL, or glycated hemoglobin levels ≥6.5%.

Abbreviations: *H. pylori*, *Helicobacter pylori*; HbA1c, hemoglobin A1c; HDL-C, high-density lipoprotein cholesterol.

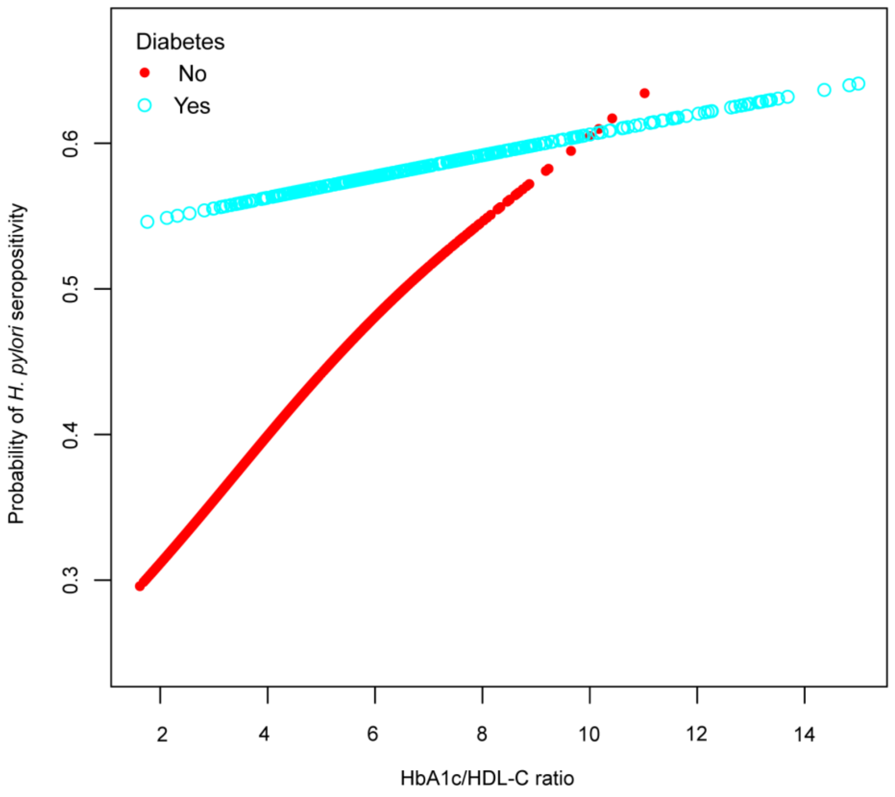

Supplementary Figure 1 The association between the HbA1c/HDL-C ratio and *H. pylori* seropositivity stratified by diabetes status

Diabetes was identified based on self-reported physician diagnosis, use of insulin or glucose-lowering medications, fasting blood glucose levels ≥126 mg/dL, or glycated hemoglobin levels ≥6.5%. The red line represents the smooth curve fit for individuals with diabetes, while the blue line represents the smooth curve fit for individuals without diabetes. The model was adjusted for age, sex, race, education, marital status, PIR, drinking, smoking, BMI, hypertension, diabetes, and cardiovascular disease.

Abbreviation: *H. pylori*, *Helicobacter pylori*; HbA1c, hemoglobin A1c; HDL-C, high-density lipoprotein cholesterol.
